# Admission Physiology Appeared More Informative Than Small-for-Gestational-Age Status for Predicting Severe In-Hospital Disposition in Preterm Infants Born at 28–36 Weeks: A Retrospective Single-Center NICU Cohort Study

**DOI:** 10.1186/s12887-026-06825-3

**Published:** 2026-04-15

**Authors:** Zeinab Ghorooneh, Ashraf Mohammadzadeh, Ahmad shah Farhat, Nafiseh Daei, Ezzat Khodashenas

**Affiliations:** Neonatal Research Center, Mashhad University of Medical Sciences, Mashhad, Iran

**Keywords:** Small for gestational age, Premature, Intensive care units, neonatal, Length of stay, Prognosis

## Abstract

**Background:**

Whether small for gestational age adds prognostic information after NICU admission physiology is known remains uncertain in preterm infants. This distinction is clinically important because SGA is an anthropometric birth-size classification rather than, by itself, a diagnosis of fetal growth restriction.

**Methods:**

We conducted a retrospective cohort study of 270 singleton infants born at 28 to 36 weeks’ gestation and admitted to a tertiary NICU in Mashhad, Iran, during 2022 to 2023. SGA was defined using INTERGROWTH-21st standards. The primary endpoint was severe unfavorable disposition, defined as transfer to another facility or in-hospital death versus healthy discharge, with discharge against medical advice excluded by endpoint definition. We compared prespecified binary logistic models containing gestational age and SGA alone or combined with admission physiologic variables, and internally validated the binary models using 1000 bootstrap resamples. A secondary logistic model described admission features associated with SGA status.

**Results:**

Seventy-eight infants (28.9%) were classified as SGA. Compared with AGA infants, SGA infants had lower platelet counts, lower red blood cell counts, higher venous PO2 values, and longer hospital stay. In the secondary SGA model, lower platelets and lower red blood cell counts were independently associated with SGA status. For the primary endpoint, 208 infants were eligible, including 31 transfer or death events. A model containing gestational age, log-transformed C-reactive protein, and venous pH showed better fit and discrimination than the gestational age plus SGA model, with optimism-corrected area under the curve 0.78, Brier score 0.11, and calibration slope 0.92. Adding SGA did not improve fit or discrimination, with likelihood-ratio *p* = 0.841 and adjusted OR 1.10, 95% CI 0.43 to 2.80. Replacing binary SGA with birth-weight z score also did not improve the model. When discharge against medical advice was included in a broader nonhealthy composite, discrimination fell substantially.

**Conclusion:**

In this single-center cohort of infants born at 28–36 weeks and admitted to a tertiary NICU in Mashhad, SGA as an anthropometric birth-size classification was associated with a distinct admission hematologic profile and longer hospital stay, but it did not improve prediction of severe hospital disposition after gestational age, admission venous pH, and CRP were considered. For this short-term disposition endpoint in this setting, admission physiology appeared more informative than birth-size category. These findings are hypothesis-generating, context-specific, and should not be extrapolated to longer-term outcomes or other NICU settings without external validation.

**Supplementary Information:**

The online version contains supplementary material available at 10.1186/s12887-026-06825-3.

## Introduction

Preterm birth remains a major determinant of neonatal morbidity and mortality, but gestational age alone does not fully capture risk at NICU admission. Birth size for gestational age is frequently used as an additional risk marker, especially when infants are classified as small for gestational age. However, SGA is an anthropometric designation based on birth-weight percentile, not a pathophysiologic diagnosis. Without antenatal Doppler studies, interval fetal growth data, or placental evaluation, SGA cannot reliably distinguish constitutionally small infants from those affected by placental dysfunction or other causes of impaired fetal growth [1]. For this reason, SGA should be interpreted as a birth-size classification rather than used interchangeably with fetal growth restriction.

Among preterm infants, SGA has been associated with a higher burden of selected short-term complications. In gestation-matched cohorts of preterm infants born between 28 and 36 weeks, SGA status has been linked to higher rates of hypoglycemia, necrotizing enterocolitis, polycythemia, late-onset sepsis, and prolonged hospitalization, while associations with respiratory distress syndrome and in-hospital mortality have been less consistent after accounting for gestational age [2]. By contrast, bronchopulmonary dysplasia has been reported more consistently in some SGA preterm cohorts, although such findings do not distinguish constitutionally small infants from infants with underlying fetal-growth pathology and should not be interpreted mechanistically in the absence of antenatal characterization [3]. Hematologic differences are also described, particularly lower platelet indices and more frequent thrombocytopenia in smaller infants and in those born after maternal hypertensive disease [4, 5]. In a large multihospital neonatal study, thrombocytopenia attributable to the SGA state alone carried low associated mortality, whereas thrombocytopenia from a recognized pathologic cause such as sepsis was associated with substantially higher case fatality [6]. Yet these associations do not establish that SGA itself remains an informative prognostic marker once the infant’s physiologic condition at admission is known.

That question is clinically relevant because bedside decision-making in the NICU is driven less by birth phenotype alone than by the infant’s current physiologic state. Admission acid-base balance, inflammatory markers, and hematologic indices may serve as more proximate markers of acute illness severity than an anthropometric label alone. Across neonatal studies, early physiologic abnormalities such as acidosis, inflammatory activation, and impaired ventilation are associated with adverse short-term outcomes beyond baseline demographic variables [7, 8]. At the same time, small-event prognostic analyses are especially vulnerable to overfitting, making parsimonious model construction and internal validation essential when testing whether admission physiology adds value beyond gestational age and birth size [9, 10].

Interpretation of the SGA literature is further complicated by measurement and classification issues. SGA prevalence varies according to the growth reference used, including INTERGROWTH-21st, Fenton, and local charts, and these differences can alter both case mix and apparent outcome associations [11, 12]. In very-low-birth-weight cohorts, the proportion classified as SGA at birth differs significantly between INTERGROWTH-21st and Fenton, and chart-dependent classification has been shown to alter associations with extrauterine growth restriction and downstream clinical courses [13]. Because SGA classification depends directly on gestational age, inaccuracies in dating can also shift infants across percentile thresholds. Dating based on last menstrual period or postnatal examination is less precise than early ultrasonography and may contribute materially to exposure misclassification [14]. Postnatal assessments such as the Ballard score can systematically overestimate gestational age in growth-restricted infants, potentially reclassifying truly SGA neonates as AGA for an erroneously higher gestational age [15]. In many low and middle income settings, discharge-based outcomes are additionally shaped by discharge against medical advice, which reflects family, financial, and health-system pressures as much as clinical trajectory [16, 17].

Evidence from Iran and the surrounding region on the joint prognostic role of SGA classification and admission physiology in preterm infants remains limited. We therefore conducted a retrospective cohort study of singleton preterm infants born at 28 to 36 weeks and admitted to a tertiary NICU in Mashhad, Iran. Our aims were to compare baseline characteristics, admission physiology, and early hospital outcomes between SGA and AGA infants, to describe early admission features associated with SGA status, and to test whether SGA classification added prognostic information for severe unfavorable hospital disposition beyond gestational age and a parsimonious set of admission physiologic variables. Because discharge against medical advice was common and clinically ambiguous, we prespecified outcome formulations that handled DAMA separately from clearly clinical adverse outcomes.

## Methods

### Study design and setting

This retrospective observational cohort study was conducted in the Neonatal Intensive Care Unit of Imam Reza Hospital, a tertiary referral center affiliated with Mashhad University of Medical Sciences, Mashhad, Iran, during 2022 to 2023. The study used routinely collected clinical data extracted from maternal and neonatal records after completion of care. No study-specific interventions, tests, or alterations in clinical management were introduced.

### Participants

All consecutive eligible preterm infants admitted to the NICU during the study period were considered for inclusion. Eligibility criteria were singleton birth and gestational age from 28 to 36 completed weeks. Infants were excluded if they had multiple gestations, major congenital malformations, chromosomal abnormalities, or large for gestational age birth weight, defined as birth weight above the 90th percentile on INTERGROWTH-21st standards. Each infant contributed one record to the analytic dataset.

Because the study was based on a fixed retrospective cohort of all eligible admissions within a defined period, no prospective sample-size calculation was used to determine enrollment. Instead, multivariable model complexity was restricted according to the frequency of the study outcomes.

### Gestational age determination and birth-size classification

Gestational age was abstracted from the maternal obstetric record and neonatal chart using a prespecified hierarchy of data sources. Early pregnancy ultrasonography was used as the primary source when documented. If early ultrasonography was unavailable, a reliable last menstrual period recorded in the obstetric chart was used. If neither source was available, or if the clinical record indicated uncertainty in obstetric dating, postnatal Ballard assessment was used as the fallback method. When more than one dating source was documented, the estimate from the highest-priority available source in the prespecified hierarchy was retained for analysis (early ultrasonography first, then reliable last menstrual period, then postnatal Ballard assessment). This refers to source priority rather than to the numerically highest gestational age estimate. In the extracted analytic dataset, gestational age was available in completed weeks only; therefore, birth-size classification could not incorporate week-plus-day resolution.

Birth weight was measured immediately after delivery using calibrated neonatal scales. Birth-size category was assigned using INTERGROWTH-21st standards for gestational age and sex. Small for gestational age was defined as birth weight below the 10th percentile, and appropriate for gestational age as birth weight from the 10th to the 90th percentile. Infants above the 90th percentile were excluded. Because antenatal Doppler data were unavailable, SGA was treated throughout as an anthropometric birth-size classification and not as a surrogate for fetal growth restriction.

### Data sources and study variables

Data were extracted retrospectively from maternal obstetric records and neonatal NICU charts using a structured abstraction form (Table 1). Study variables were grouped a priori into baseline maternal and perinatal characteristics, admission physiologic variables, and post-admission outcomes.

**Table 1:** Study variables and operational definitions

Domain	Variable	Operational definition	Unit or coding	Role in analyses
Birth-size classification	SGA status	Birth weight below the 10th percentile for gestational age and sex using INTERGROWTH-21st standards	SGA or AGA	Main exposure
Baseline perinatal	Gestational age	Obstetric age at birth determined using hierarchical dating from early ultrasonography, reliable last menstrual period, or Ballard assessment	Completed weeks	Baseline covariate
Baseline perinatal	Birth weight	Weight measured immediately after delivery on calibrated neonatal scales	g	Descriptive variable and sensitivity birth-size coding
Baseline maternal	Maternal age	Maternal age at delivery	years	Baseline covariate
Baseline neonatal	Infant sex	Sex recorded at birth	Male or female	Baseline covariate
Baseline obstetric	Mode of delivery	Route of delivery recorded in the obstetric chart	Cesarean or vaginal	Baseline covariate
Baseline obstetric	Maternal hypertension	Hypertensive disorder documented by the treating obstetric team during pregnancy	Yes or no	Baseline covariate
Baseline obstetric	Gestational diabetes	Gestational diabetes documented in the obstetric record	Yes or no	Baseline covariate
Baseline obstetric	Premature rupture of membranes	Rupture of membranes before labor as recorded in the maternal chart	Yes or no	Baseline covariate
Baseline obstetric	Chorioamnionitis	Clinical diagnosis documented before or at delivery	Yes or no	Baseline covariate
Baseline obstetric	Urinary tract infection	Maternal urinary tract infection recorded during pregnancy	Yes or no	Baseline covariate
Baseline neonatal	Apgar score	Standard Apgar assessment after birth	1-minute and 5-minute scores	Descriptive variable
Admission physiology	Hemoglobin	Admission hemoglobin concentration	g/dL	Descriptive and screening variable
Admission physiology	Platelet count	Admission platelet count from complete blood count	×10³/µL	Secondary SGA model
Admission physiology	White blood cell count	Admission white blood cell count	×10³/µL	Descriptive and screening variable
Admission physiology	Red blood cell count	Admission red blood cell count	×10⁶/µL	Secondary SGA model and sensitivity outcome model
Admission physiology	C-reactive protein	Admission CRP concentration	mg/L	Primary outcome model after log transformation
Admission physiology	Venous pH	Admission venous blood gas pH	Unitless	Primary outcome model
Admission physiology	Venous PCO₂	Admission venous partial pressure of carbon dioxide	mmHg	Screening and sensitivity analysis
Admission physiology	Venous HCO₃	Admission venous bicarbonate concentration	mEq/L	Descriptive and screening variable
Admission physiology	Venous PO₂	Admission venous partial pressure of oxygen	mmHg	Secondary SGA model
Post-admission outcome	Respiratory distress syndrome	Diagnosis recorded by the treating neonatal team after admission	Yes or no	Descriptive post-admission outcome
Post-admission outcome	Length of hospital stay	Time from birth to final hospital disposition	Days	Descriptive post-admission outcome
Final disposition	Healthy discharge	Discharge in good general condition	Yes or no within multicategory outcome	Reference outcome
Final disposition	Discharge against medical advice	Self-discharge before medically recommended discharge	Yes or no within multicategory outcome	Secondary and sensitivity endpoint component
Final disposition	Transfer	Transfer to another facility before final discharge	Yes or no within multicategory outcome	Primary endpoint component
Final disposition	Death	In-hospital death before discharge	Yes or no within multicategory outcome	Primary endpoint component

Baseline variables included infant sex, mode of delivery, gestational age, birth weight, maternal age, Apgar scores at 1 and 5 min, and maternal conditions documented during pregnancy or at delivery, including hypertension, gestational diabetes, premature rupture of membranes, chorioamnionitis, and urinary tract infection.

Admission physiologic variables comprised complete blood count indices, serum C-reactive protein, and venous blood gas measurements obtained as part of routine NICU assessment. The prespecified admission laboratory variables were hemoglobin, platelet count, white blood cell count, red blood cell count, CRP, venous pH, venous partial pressure of carbon dioxide, venous bicarbonate, and venous partial pressure of oxygen. These samples were obtained from peripheral venous blood, usually within the first 6 h after NICU admission according to unit practice. The exact postnatal age at sampling was not recorded consistently enough to be analyzed as a covariate.

Post-admission outcomes included respiratory distress syndrome, length of hospital stay, and final hospital disposition. Final disposition was categorized as healthy discharge, discharge against medical advice, transfer to another facility, or in-hospital death. Length of stay was defined as the interval from birth to final hospital disposition.

### Outcome definitions

The primary inferential endpoint was severe unfavorable disposition, defined as transfer to another facility or in-hospital death, with healthy discharge as the reference category. Infants discharged against medical advice were excluded from this primary endpoint by design because their subsequent clinical course was unknown and because DAMA may reflect social and financial determinants in addition to clinical status.

Secondary and sensitivity outcome formulations were also examined. These included a broader nonhealthy composite defined as DAMA, transfer, or death versus healthy discharge, a best-case sensitivity analysis in which all DAMA cases were reclassified as healthy discharge, and an exploratory four-category disposition analysis that retained all discharge categories. In addition, a secondary associational analysis modeled SGA status as the dependent variable in order to describe early admission features associated with anthropometric birth-size classification at admission.

### Statistical analysis

Descriptive analyses were performed using IBM SPSS Statistics version 25. Regression modeling, bootstrap validation, and graphical analyses were performed in R version 4.5.1.

The outcome definitions, descriptive comparisons, and limited set of fixed candidate regression models were specified before the final model refitting reported here. Ategorical variables were summarized as counts and percentages. Continuous variables were summarized primarily as median and interquartile range. For supplementary four-category outcome summaries, approximately symmetric continuous variables were additionally described by mean and standard deviation to facilitate inspection of group patterns.

For comparisons between SGA and AGA infants, categorical variables were analyzed using Fisher exact tests and continuous variables using Wilcoxon rank-sum tests. For univariable screening across the four discharge categories, continuous variables were analyzed using Kruskal-Wallis tests and categorical variables using Fisher-Freeman-Halton exact tests with Monte Carlo estimation when required by table size. Effect sizes were reported as rank-biserial correlation for Wilcoxon tests, epsilon-squared for Kruskal-Wallis tests, and Cramer’s V for categorical analyses.

To limit false-positive findings in descriptive screening, Benjamini-Hochberg false discovery rate control was applied within each prespecified family of related comparisons. These families corresponded to baseline categorical characteristics, baseline continuous characteristics, admission physiologic variables, post-admission outcomes by SGA group, and four-category disposition screening sets. All hypothesis tests were 2 sided, and effect estimates were reported with 95% confidence intervals where applicable.

### Secondary logistic model for SGA classification

A secondary multivariable logistic regression model was fitted with SGA status as the dependent variable to characterize early admission features associated with anthropometric birth-size category. To preserve parsimony, the candidate covariates were restricted to gestational age, platelet count, red blood cell count, venous PO2, maternal hypertension, and gestational diabetes. For interpretability, platelet count was scaled per 50 × 10^9/L, red blood cell count per 1 × 10^6/µL, and venous PO2 per 10 mmHg. An RBC by PO2 interaction term was assessed only in an exploratory analysis. Because sparse-data bias was a potential concern in this secondary model, a Firth penalized logistic regression analysis was also performed as a robustness check.

### Primary multivariable outcome modeling

Because transfer and death were uncommon as separate outcome categories, the primary multivariable analysis used binary logistic regression for severe unfavorable disposition, defined as transfer or death versus healthy discharge, with DAMA excluded by endpoint definition. To avoid overfitting, the primary analysis did not use a high-dimensional multinomial model or data-driven stepwise selection. Instead, a small set of fixed candidate models was compared to test whether early physiologic measurements added information beyond gestational age and anthropometric birth-size category.

Three binary logistic models were prespecified for the primary endpoint. Model A included gestational age and SGA status. Model B included gestational age, log-transformed CRP, and venous pH. Model C included gestational age, SGA status, log-transformed CRP, and venous pH. A further sensitivity model added red blood cell count to Model C because of its biological plausibility and its association in univariable screening.

CRP was transformed as log(CRP + 1) to reduce right skew. Venous pH was scaled per 0.1-unit increase. Continuous terms in the primary outcome analysis were modeled as linear terms because the limited number of events did not support flexible spline-based modeling. Incremental model value was examined using nested likelihood-ratio tests and Akaike information criterion. Adjusted odds ratios with 95% confidence intervals were reported.

### Sensitivity and exploratory analyses

Several sensitivity analyses were conducted. First, binary SGA status was replaced by continuous birth-weight z score in the primary severe unfavorable disposition model. Second, DAMA handling was examined by refitting the reduced outcome models to the nonhealthy composite and to the best-case scenario in which all DAMA cases were reclassified as healthy discharge. Third, venous PCO2 was assessed as an add-on term to the combined model and retained only if it materially improved model fit.

Finally, an exploratory reduced multinomial logistic model was fitted for the four-category disposition outcome using a limited predictor set consisting of gestational age, SGA status, red blood cell count, venous pH, logCRP, and venous PCO2. Because the transfer and death categories were sparse, this multinomial analysis was treated as exploratory. Accordingly, inference from that model was limited to global drop-one likelihood-ratio tests and relative risk ratio point estimates, without category-specific Wald confidence intervals.

### Internal validation and model performance

Internal validation of each fixed binary outcome model was performed using 1000 bootstrap resamples to estimate optimism-corrected performance. Reported model-performance measures were the area under the receiver operating characteristic curve, Brier score, calibration intercept, and calibration slope. Because model choice itself can introduce optimism, an additional selection-aware bootstrap procedure was performed across the candidate binary models to quantify model-selection uncertainty and process-level overfitting. In each bootstrap resample, the full candidate-model comparison procedure was repeated, the selected model was refit within that resample, and performance was then evaluated in both the bootstrap and original samples; the average difference was used to estimate optimism from the overall model-selection process rather than from a single fixed model. Calibration plots based on grouped predicted risks were used descriptively only.

For the exploratory multinomial model, supplementary performance summaries included multiclass Brier score and one-versus-rest area under the curve values.

### Missing data

Data completeness was audited for all variables considered in descriptive and multivariable analyses. No imputation was performed. Analyses used complete-case data, and the analytic denominator for each model was reported explicitly. Differences in analytic sample size across models therefore reflected endpoint definition and model eligibility rather than imputation procedures.

### Ethics

This retrospective cohort study was approved by the Research Ethics Committee of Mashhad University of Medical Sciences (approval code: IR.MUMS.MEDICAL.REC.1401.257). The study was conducted in accordance with the Declaration of Helsinki and its later amendments. Because this study used de-identified routinely collected clinical data, involved no direct contact with participants, and introduced no study-specific intervention, the requirement for informed consent to participate from the infants’ parents or legal guardians was waived by the Research Ethics Committee of Mashhad University of Medical Sciences.

## Results

### Study population and data completeness

A total of 270 preterm infants met the eligibility criteria and were included in the analysis. Of these, 192 infants (71.1%) were classified as appropriate for gestational age and 78 (28.9%) as small for gestational age. Male infants accounted for 156 of 270 infants (57.8%). All recorded variables used in the descriptive and multivariable analyses were complete in the available dataset, so no infant was excluded because of missing data. Gestational age was available at week-level resolution only in the dataset used for reanalysis.

### Baseline maternal, perinatal, and birth characteristics

Baseline maternal, perinatal, and birth characteristics are summarized in Table 2. Median gestational age was similar in the two groups, 33.5 [IQR 4.0] weeks in the AGA group and 33.0 [IQR 2.75] weeks in the SGA group (*p* = 0.470). Maternal age, Apgar scores, sex distribution, mode of delivery, maternal hypertension, gestational diabetes, premature rupture of membranes, chorioamnionitis, and urinary tract infection did not differ significantly between groups. As expected from the exposure definition, birth weight was lower in the SGA group.

**Table 2:** Maternal, perinatal, and birth characteristics by SGA status

Variable	AGA (*n* = 192)	SGA (*n* = 78)	*P* value
Male sex, n (%)	114 (59.4)	42 (53.8)	0.418
Cesarean delivery, n (%)	155 (80.7)	63 (80.8)	1.000
Maternal hypertension, n (%)	16 (8.3)	13 (16.7)	0.053
Gestational diabetes, n (%)	37 (19.3)	9 (11.5)	0.154
Premature rupture of membranes, n (%)	17 (8.9)	4 (5.1)	0.452
Chorioamnionitis, n (%)	1 (0.5)	3 (3.8)	0.074
Urinary tract infection, n (%)	3 (1.6)	2 (2.6)	0.629
Gestational age, weeks	33.5 [4.0]	33.0 [2.75]	0.470
Birth weight, g	2060 [837.5]	1280 [467.5]	< 0.001
Maternal age, years	34.0 [10.0]	32.5 [12.75]	0.178
Apgar score at 1 min	8.0 [3.0]	8.0 [3.0]	0.401
Apgar score at 5 min	9.0 [2.0]	9.0 [2.0]	0.888

Continuous variables are shown as median [IQR]. Birth-weight comparison is descriptive because SGA classification is defined from birth weight and gestational age

### Admission physiology

Admission laboratory and venous blood gas findings are shown in Table 3. After false discovery rate correction within this family of comparisons, SGA infants had lower platelet counts, lower RBC counts, and higher venous PO₂ than AGA infants. CRP was higher in the SGA group before correction but did not remain below the false discovery rate threshold after correction. Hemoglobin, white blood cell count, venous pH, venous PCO₂, and bicarbonate were similar between groups.

**Table 3:** Admission laboratory and venous blood gas findings by SGA status

Variable	AGA (*n* = 192)	SGA (*n* = 78)	*P* value	FDR q
Hemoglobin, g/dL	17.10 [3.85]	17.10 [3.55]	0.849	0.849
Platelets, ×10³/µL	234.5 [98.5]	185.5 [111.5]	< 0.001	< 0.001
White blood cells, ×10³/µL	12.20 [7.55]	11.75 [8.78]	0.536	0.784
Red blood cells, ×10⁶/µL	4.70 [1.00]	4.39 [0.89]	0.008	0.036
CRP, mg/L	1.00 [2.90]	1.35 [5.18]	0.047	0.106
Venous pH	7.27 [0.09]	7.28 [0.11]	0.584	0.784
Venous PCO₂, mmHg	43.6 [14.35]	43.6 [13.40]	0.722	0.812
Venous HCO₃, mEq/L	20.45 [5.55]	20.03 [4.33]	0.610	0.784
Venous PO₂, mmHg	51.5 [18.10]	58.97 [18.82]	0.012	0.036

Values are median [IQR]. FDR q values were calculated within this admission-physiology comparison family

In a secondary logistic model of SGA classification that included all 270 infants, lower platelet count and lower RBC count were associated with SGA status. The adjusted odds ratios were 1.11 per week for gestational age (95% CI 0.97 to 1.26, *p* = 0.138), 0.71 per 50 × 10⁹/L for platelets (95% CI 0.59 to 0.86, *p* < 0.001), 0.57 per 1 × 10⁶/µL for RBC (95% CI 0.38 to 0.86, *p* = 0.007), 1.13 per 10 mmHg for venous PO₂ (95% CI 0.98 to 1.30, *p* = 0.091), 2.21 for maternal hypertension (95% CI 0.96 to 5.13, *p* = 0.064), and 0.64 for gestational diabetes (95% CI 0.28 to 1.46, *p* = 0.286). An exploratory RBC and PO₂ interaction did not meet conventional significance criteria (OR 1.23, 95% CI 0.97 to 1.55, *p* = 0.082; likelihood-ratio *p* = 0.073). Full secondary-model results, conditional interaction estimates, and Firth sensitivity analyses are presented in Supplementary Tables S4 to S6.

### Post-admission outcomes by SGA group

Post-admission outcomes are summarized in Table 4. Respiratory distress syndrome was less frequent in SGA infants than in AGA infants, 48.7% versus 62.5%, but this difference did not remain below the false discovery rate threshold after correction. Length of stay was longer in the SGA group, with a median of 8.5 [IQR 12.75] days versus 6.0 [IQR 6.0] days in the AGA group. The overall four-category discharge distribution did not differ by SGA status.

**Table 4:** Post-admission outcomes by SGA status

Variable		AGA (*n* = 192)	SGA (*n* = 78)	*P* value	FDR q
Respiratory distress syndrome, n (%)		120 (62.5)	38 (48.7)	0.037	0.056
Length of stay, days		6.0 [6.0]	8.5 [12.75]	0.009	0.027
Discharge disposition, overall test	Healthy discharge, n (%)	126 (65.6)	51 (65.4)	0.685	0.685
	DAMA, n (%)	46 (24.0)	16 (20.5)		
	Transfer, n (%)	6 (3.1)	2 (2.6)		
	Death, n (%)	14 (7.3)	9 (11.5)		

Length of stay is shown as median [IQR]. FDR q values were calculated within the post-admission outcome family in this table

### Univariable screening across four discharge categories

Across the full cohort, discharge outcomes were healthy discharge in 177 infants (65.6%), DAMA in 62 (23.0%), transfer in 8 (3.0%), and death in 23 (8.5%). In the prespecified univariable screening analysis across these four categories, gestational age, hemoglobin, RBC, CRP, venous pH, venous PCO₂, and maternal age were associated with outcome after false discovery rate correction (Table 5). Platelet count, white blood cell count, bicarbonate, venous PO₂, SGA status, infant sex, delivery mode, and recorded maternal comorbidities were not. Detailed per-category summaries are shown in Supplementary Tables S2 and S3.

**Table 5:** Univariable screening across four discharge categories

Variable	Effect size	*P* value	FDR q
Gestational age^a^	0.058	< 0.001	0.002
Hemoglobin^a^	0.028	0.014	0.027
Platelets^a^	0.015	0.073	0.089
White blood cells^a^	0.000	0.899	0.899
Red blood cells^a^	0.048	0.001	0.004
CRP^a^	0.028	0.015	0.027
Venous pH^a^	0.060	< 0.001	0.002
Venous PCO₂^a^	0.038	0.005	0.012
Venous HCO₃^a^	0.007	0.180	0.198
Venous PO₂^a^	0.016	0.068	0.089
Maternal age^a^	0.026	0.020	0.031
SGA status^b^	0.075	0.686	0.914
Infant sex^b^	0.036	0.950	0.990
Delivery mode^b^	0.039	0.990	0.990
Maternal hypertension^b^	0.160	0.071	0.383
Gestational diabetes^b^	0.094	0.565	0.904
Premature rupture of membranes^b^	0.154	0.096	0.383
Chorioamnionitis^b^	0.094	0.314	0.815
Urinary tract infection^b^	0.092	0.407	0.815

Effect size is epsilon-squared for Kruskal-Wallis tests and Cramer’s V for categorical tests

Tests: ^a^Kruskal-Wallis, ^b^Fisher-Freeman-Halton

### Primary multivariable outcome models

Because transfer and death were sparse as separate categories, the primary inferential endpoint was a severe unfavorable disposition composite defined as transfer or death versus healthy discharge. DAMA was excluded from this primary endpoint by design rather than treated as missing, leaving 208 infants for the primary multivariable analysis, with 31 events and no missing predictor data. Table 6 shows the candidate binary logistic models and internal bootstrap validation. In the phenotype-only model, lower gestational age was associated with severe unfavorable disposition whereas SGA was not. In the reduced physiology model, lower gestational age, higher logCRP, and lower venous pH were associated with the endpoint. Adding SGA status to this reduced physiology model did not materially change the physiologic coefficients, did not improve model fit (likelihood-ratio *p* = 0.841), and yielded a null SGA estimate (OR 1.10, 95% CI 0.43 to 2.80, *p* = 0.841). By contrast, adding physiology to the gestational-age plus SGA model improved fit (likelihood-ratio *p* = 0.0029). Among the fixed candidate models, the reduced physiology model had the lowest AIC and an optimism-corrected AUC of 0.78, with corrected Brier score 0.11 and corrected calibration slope 0.92.

**Table 6:** Binary logistic models for severe unfavorable disposition

Model	Predictor	OR	95% CI	*P* value	AIC	Corrected AUC	Corrected Brier	Corrected calibration slope
Gestational age plus SGA	Gestational age, per week	0.67	0.57 to 0.80	< 0.001	157.06	0.71	0.11	1.00
	SGA status	1.27	0.53 to 3.02	0.593				
Gestational age plus logCRP plus pH	Gestational age, per week	0.74	0.62 to 0.89	0.002	147.41	0.78	0.11	0.92
	logCRP	1.64	1.12 to 2.42	0.012				
	Venous pH, per 0.1 increase	0.49	0.27 to 0.88	0.018				
Gestational age plus SGA plus logCRP plus pH	Gestational age, per week	0.74	0.62 to 0.90	0.002	149.36	0.77	0.11	0.88
	SGA status	1.10	0.43 to 2.80	0.841				
	logCRP	1.63	1.10 to 2.42	0.016				
	Venous pH, per 0.1 increase	0.49	0.27 to 0.88	0.017				
Model comparison	Add logCRP and pH to gestational age plus SGA			0.0029				
	Add SGA to gestational age plus logCRP plus pH			0.8412				

All models used the same complete-case sample of 208 infants, including 177 healthy discharges and 31 transfer or death events. DAMA cases were excluded by endpoint definition. Corrected performance metrics are optimism-corrected bootstrap estimates from 1000 resamples. logCRP denotes log (CRP + 1)

Sensitivity analyses were consistent with the primary analysis. Replacing binary SGA with continuous birth-weight z score did not improve the primary model (OR 0.86, 95% CI 0.62 to 1.20, *p* = 0.373; likelihood-ratio *p* = 0.377). Adding RBC to the combined model did not materially improve fit (*p* = 0.155), and adding PCO₂ to the combined model did not improve fit (*p* = 0.981). When DAMA was included in a broader nonhealthy composite, discrimination was lower than in the primary DAMA-excluded analysis, with optimism-corrected AUC 0.58 for the reduced physiology model. When all DAMA cases were instead reclassified as healthy discharge, the same physiologic variables remained associated with the endpoint, and SGA status again did not add prognostic information. These sensitivity analyses, together with the reduced exploratory multinomial model, are presented in Supplementary Tables S7 to S9.

## Discussion

### Principal findings

In this cohort of 270 preterm infants born at 28 to 36 weeks, SGA status identified a distinct admission profile characterized by lower platelet counts, lower red blood cell counts, higher venous PO2 values, and longer hospital stay. However, when the outcome of interest was restricted to severe unfavorable disposition, defined as transfer or in-hospital death versus healthy discharge, SGA did not provide incremental prognostic information beyond gestational age and a small set of admission physiologic variables. A reduced model containing gestational age, venous pH, and log-transformed CRP fit the data better than a model based on gestational age and SGA alone, and adding SGA to that physiology-based model did not improve fit or discrimination. This pattern persisted when binary SGA was replaced by continuous birth-weight z score. At the same time, inclusion of DAMA in a broader nonhealthy composite substantially worsened discrimination, reinforcing that discharge outcomes in this setting are shaped by social as well as clinical processes.

The central inference, therefore, is not that birth size is biologically unimportant. Rather, it is that in this retrospective NICU cohort, SGA as an anthropometric classification did not add short-term prognostic information for severe hospital disposition once gestational maturity and current illness severity at admission were taken into account. That interpretation is consistent with the fact that SGA is a heterogeneous size-based grouping, not a uniform pathophysiologic entity [1]. Notably, large multicenter analyses have shown that the risk profile of an SGA preterm infant approximates that of an AGA infant born two to three weeks more prematurely, framing SGA as a ‘risk multiplier’ that depletes physiologic reserves beyond what gestational age alone indicates [18].

### SGA classification, prevalence, and exposure heterogeneity

Our SGA prevalence of 28.9% is higher than that reported in many preterm cohorts. Several factors may account for this. First, SGA prevalence varies meaningfully by growth reference. INTERGROWTH-21st and Fenton do not classify identical infants as below the 10th percentile, and the choice of standard can materially change both prevalence and downstream risk estimates [11, 12]. Second, our analytic dataset contained gestational age in completed weeks only, which inevitably reduces precision near percentile cut points. Third, gestational age determination in retrospective routine-care datasets is unavoidably heterogeneous, even with an explicit hierarchy, and discrepancies among early ultrasonography, last menstrual period, and postnatal assessment can shift infants across SGA thresholds [14]. These classification issues are not trivial. If infants near the 10th percentile are misclassified, true associations between birth size and outcomes may be diluted, exaggerated, or rendered unstable.

The null incremental effect of SGA in our multivariable outcome model should also be viewed through the lens of exposure heterogeneity. In the absence of antenatal Doppler data, our SGA group necessarily mixed constitutionally small infants with infants who may have had placental insufficiency, maternal vascular disease, or other forms of impaired fetal growth. That heterogeneity reduces the likelihood that a single binary birth-size label will carry strong independent prognostic information for a short-term clinical endpoint once more proximate physiologic measures are included [1].

### Admission physiology and short-term prognosis

The best-performing primary model contained gestational age, venous pH, and CRP. These variables are clinically plausible markers of immediate illness severity. Lower gestational age captures baseline developmental vulnerability. Lower pH reflects acute acid-base derangement, which may arise from respiratory compromise, poor perfusion, or mixed systemic stress. Higher CRP likely reflects inflammatory activation, whether infectious or noninfectious, at the time of presentation. Together, these measures appeared, in this cohort, to summarize short-term physiologic instability more effectively than birth-size category alone for this specific disposition-based endpoint. This interpretation is aligned with broader neonatal literature showing that early physiologic abnormalities improve short-term prognostic discrimination beyond baseline perinatal descriptors [7, 8].

Importantly, these admission variables should be interpreted as contemporaneous severity markers rather than mechanistic mediators of SGA. Our data do not support causal claims that acidosis or inflammation explain why an infant was born SGA, nor do they justify interventional recommendations based on observational associations. They indicate only that, at NICU admission, pH and CRP conveyed more information about the risk of transfer or death than SGA status did.

The model’s optimism-corrected AUC of 0.78 and calibration slope of 0.92 are encouraging for a low-dimensional retrospective analysis, but they should not be overstated. These values indicate moderate internal performance after bootstrap correction, not a ready-for-use clinical prediction tool. Contemporary guidance for small-event prognostic studies emphasizes restrained model complexity, internal validation, and caution in interpretation, especially before any external validation is available [9, 10].

### Hematologic features associated with SGA at admission

The secondary model describing admission features associated with SGA identified lower platelet count and lower red blood cell count as the strongest correlates. The platelet finding is concordant with prior neonatal studies in which smaller infants, especially those born after maternal hypertensive disease, had lower platelet counts or platelet indices at birth and during the first days of life [4, 5]. https://pmc.ncbi.nlm.nih.gov/articles/PMC4906543/ In our study, this should be understood only as an early admission feature associated with anthropometric smallness, not as a causal antecedent or a basis for platelet-directed clinical intervention. Prior neonatal reports have described early thrombocytopenia in some SGA infants with a clinical course distinct from thrombocytopenia associated with recognized pathologic causes such as sepsis or disseminated intravascular coagulation; however, our dataset was not designed to characterize platelet trajectories over time [6].

The lower red blood cell counts in our SGA group are less consistent with literature emphasizing polycythemia in some growth-restricted populations. However, polycythemia is not universal in preterm SGA cohorts, and absolute red cell indices vary with gestational age, timing of sampling, capillary versus venous measurement, maternal hematologic status, early fluid shifts, and the proportion of constitutionally small infants within the SGA group [19, 20]. Because our dataset lacked precise postnatal sampling time, reticulocyte counts, nucleated red blood cells, hematocrit, and placental data, the RBC result should be interpreted descriptively rather than mechanistically.

The higher venous PO2 observed in SGA infants also warrants restraint. This difference was present in unadjusted comparisons but weakened in multivariable modeling, and venous PO2 is an imprecise and context-sensitive indicator that depends on sampling conditions, perfusion, respiratory support, and local extraction. The exploratory RBC by PO2 interaction did not meet conventional significance criteria, and we do not attach clinical or mechanistic meaning to it. In particular, these data do not justify any recommendation regarding oxygen targets.

### Relation to prior outcome studies in preterm SGA infants

Our results fit a pattern seen in several contemporary cohorts of preterm infants between 28 and 36 weeks. In GA-matched analyses, SGA has been associated more consistently with specific metabolic, gastrointestinal, infectious, and hematologic morbidities and with prolonged hospitalization than with RDS or in-hospital mortality per se [2]. In our data, respiratory distress syndrome was less frequent in SGA infants in the raw comparison, but the difference did not remain below the false discovery threshold. This pattern is directionally similar to some prior cohort reports in which SGA status showed different associations with acute respiratory diagnoses and later pulmonary morbidity, although those studies involved different populations and endpoints and should not be interpreted as mechanistic explanations for our findings [3]. Likewise, the overall four-category discharge distribution did not differ by SGA status. These observations are more consistent with a heterogeneous short-term SGA phenotype than with a uniform excess risk across all hospital outcomes. In larger datasets with sufficient events, however, SGA status has been independently associated with composite morbidity and mortality in preterm infants, with a clear dose-response gradient below the 3rd percentile [21], suggesting that null findings in smaller cohorts may partly reflect limited statistical power or endpoint-specific effects.

The longer length of stay in SGA infants in our cohort also accords with earlier work showing that smaller preterm infants often require more prolonged hospitalization [2]. However, our analysis was not designed to explain why. We did not model LOS using adjusted time-to-event methods, and we did not collect a sufficiently granular set of downstream complications such as hypoglycemia burden, enteral feeding progression, catheter days, or culture-confirmed late sepsis. Consequently, we cannot determine whether the longer stay reflected slower physiologic stabilization, greater feeding difficulty, more frequent infection evaluation, or other unmeasured processes.

The null result for continuous birth-weight z score is also worth noting. In larger datasets, continuous size measures often outperform dichotomous SGA thresholds by preserving information across the full distribution [22]. In our cohort, however, replacing SGA with birth-weight z score did not improve the severe-outcome model. This suggests that the absence of incremental prognostic value was not merely an artifact of dichotomization. Rather, for this short-term endpoint, size at birth, however parameterized, contributed less information than gestational age and immediate admission physiology.

### DAMA and the interpretation of discharge-based outcomes

One of the most important methodological lessons from this study concerns DAMA. Our DAMA rate of 23.0% is high, but it is not unusual in comparable settings. Recent NICU reports from Iran and Nepal describe DAMA rates in a similar range and identify family pressures, dissatisfaction, financial constraints, and prolonged hospitalization as major drivers [16, 17]. This is why we excluded DAMA from the primary severe unfavorable disposition endpoint by design rather than treating it as missing at random or folding it automatically into an adverse clinical composite.

The sensitivity analyses validated that choice. When DAMA was included in a broader nonhealthy composite, optimism-corrected discrimination fell substantially. By contrast, when DAMA cases were reclassified in a best-case manner as healthy discharge, the same physiologic variables remained associated with outcome and SGA remained uninformative. The implication is that DAMA behaves differently from transfer or death. It is a mixed social and clinical endpoint. Its inclusion weakens the interpretability and transportability of any model intended to represent illness trajectory.

This matters for generalizability. In settings with lower DAMA rates, discharge disposition may more closely reflect clinical evolution. In our setting, however, a sizable proportion of final disposition was shaped by factors that were not recorded in the dataset, including likely socioeconomic stress and family-level decision making. Other NICU network data suggest that many DAMA infants remain clinically unstable at departure and may face substantial post-discharge mortality [23]. Because we lacked post-discharge follow-up, the true adverse burden after DAMA could not be quantified. In resource-limited settings more broadly, the combination of prematurity and SGA has been associated with markedly elevated neonatal mortality, with pooled estimates suggesting up to a 15-fold increase in the risk of death compared with term AGA infants [24]; unmeasured post-DAMA mortality may further amplify this burden in cohorts like ours. These findings pertain to short-term hospital disposition only and should not be extrapolated to neurodevelopmental, post-discharge growth, or later respiratory outcomes, for which SGA may still retain independent prognostic relevance.

### Strengths and limitations

This study has several strengths. We explicitly redefined the design as a retrospective cohort study and aligned the analysis with a clinically interpretable prognostic question. We prespecified parsimonious fixed models for the primary endpoint rather than repeating a high-dimensional multinomial strategy unsuited to the observed event counts. We reported model-specific denominators, avoided imputation where no missingness existed, and used bootstrap internal validation to obtain optimism-corrected estimates of discrimination and calibration. We also examined the robustness of the findings to alternative birth-size coding and to different DAMA-handling strategies.

The limitations are equally important. This was a single-center retrospective study, and the primary outcome analysis contained only 31 transfer or death events. Although the modeling strategy was deliberately simplified, the estimates remain vulnerable to imprecision and should be regarded as exploratory until externally validated. Gestational age was available only in completed weeks, and classification error related to dating source or birth-size standard may have affected the exposure definition. Because antenatal Doppler studies were unavailable, SGA could not be partitioned into constitutionally small and pathologically growth-restricted subgroups. Admission laboratory sampling was usually performed within the first hours after admission, but the exact postnatal timing was not consistently recorded, which limits interpretation of hematologic and blood-gas measures. Venous rather than arterial gas data were available, and we lacked concurrent SpO2 and FiO2 values. Finally, we had no socioeconomic follow-up data to model DAMA determinants or post-discharge outcomes.

## Conclusion

In this retrospective single-center cohort of preterm infants born at 28–36 weeks and admitted to a tertiary NICU in Mashhad, SGA as an anthropometric birth-size classification was associated with lower platelet and red blood cell counts at admission and with longer hospital stay, but it did not improve prediction of severe unfavorable hospital disposition once gestational age, venous pH, and CRP were considered. For this short-term disposition endpoint in this setting, admission physiology appeared more informative than birth-size classification. These findings should be interpreted within the context of this regional tertiary NICU cohort, the endpoint definition that excluded DAMA, and the absence of antenatal Doppler characterization. They do not address longer-term neurodevelopmental or growth outcomes, and they require external validation before any broader prognostic claims are made.

## Supplementary Information

Supplementary Material 1.

## Data Availability

The dataset supporting this article’s conclusions is confidential and cannot be made publicly available. However, data may be available upon reasonable request and with permission of the original data providers, subject to compliance with applicable confidentiality agreements and data protection laws, via contact through the Neonatal Research Center at Mashhad University of Medical Sciences, at the following email address: KhodashenasE@mums.ac.ir
