# Supplementary material for: Admission Physiology Appeared More Informative Than Small-for-Gestational-Age Status for Predicting Severe In-Hospital Disposition in Preterm Infants Born at 28–36 Weeks: A Retrospective Single-Center NICU Cohort Study
Source: BMC Pediatr. 2026 Apr 15;26:493. doi: 10.1186/s12887-026-06825-3 (PMC13214418; doi:10.1186/s12887-026-06825-3)
Supplement: Supplementary file 1 — Supplementary Material 1. [file 12887_2026_6825_MOESM1_ESM.docx]

**Supplementary Appendix**

All supplementary analyses used the same cohort of 270 infants. No recorded variable used in these analyses had missing data. Differences in analytic sample size arose only from endpoint definition.

Table 1. Analytic samples and endpoint definitions

| **Analysis** | **Starting N** | **Complete-case N** | **Outcome or class counts** |
| --- | --- | --- | --- |
| SGA group comparisons | 270 | 270 | AGA 192, SGA 78 |
| Secondary logistic model for SGA classification | 270 | 270 | AGA 192, SGA 78 |
| Four-category discharge screening | 270 | 270 | Healthy 177, DAMA 62, Transfer 8, Death 23 |
| Primary severe unfavorable disposition model | 208 | 208 | Healthy 177, Transfer or death 31, DAMA excluded by design 62 |
| Birth-weight z-score sensitivity | 208 | 208 | Healthy 177, Transfer or death 31, DAMA excluded by design 62 |
| Nonhealthy composite sensitivity | 270 | 270 | Healthy 177, DAMA or transfer or death 93 |
| Best-case DAMA healthy sensitivity | 270 | 270 | Healthy 239, Transfer or death 31 |
| Exploratory reduced multinomial model | 270 | 270 | Healthy 177, DAMA 62, Transfer 8, Death 23 |

DAMA exclusion in the primary endpoint was a structural endpoint definition and not missing data.

Table 2. Continuous variables by four-category discharge disposition

| **Variable** | **Healthy discharge (n=177)** | **DAMA (n=62)** | **Transfer (n=8)** | **Death (n=23)** | **P value** | **FDR q** |
| --- | --- | --- | --- | --- | --- | --- |
| Gestational age, weeks | 33.15 ± 2.08 | 33.37 ± 2.03 | 31.25 ± 2.87 | 30.70 ± 3.11 | <0.001 | 0.002 |
| Hemoglobin, g/dL | 17.22 ± 3.11 | 17.70 ± 2.69 | 15.28 ± 1.79 | 15.98 ± 2.62 | 0.014 | 0.027 |
| Platelets, ×10³/μL | 234.0 [101.0] | 220.5 [121.0] | 200.5 [97.25] | 190.0 [96.0] | 0.073 | 0.089 |
| White blood cells, ×10³/μL | 13.27 ± 6.26 | 12.66 ± 4.54 | 13.23 ± 4.56 | 14.51 ± 7.64 | 0.899 | 0.899 |
| Red blood cells, ×10⁶/μL | 4.68 ± 0.80 | 4.71 ± 0.75 | 4.22 ± 0.41 | 4.05 ± 0.68 | 0.001 | 0.004 |
| CRP, mg/L | 0.92 [3.00] | 1.30 [2.88] | 3.70 [6.67] | 2.60 [6.27] | 0.015 | 0.027 |
| Venous pH | 7.269 ± 0.073 | 7.287 ± 0.068 | 7.218 ± 0.053 | 7.220 ± 0.077 | <0.001 | 0.002 |
| Venous PCO₂, mmHg | 43.68 [13.30] | 39.15 [11.68] | 56.30 [11.90] | 47.00 [15.75] | 0.005 | 0.012 |
| Venous HCO₃, mEq/L | 20.70 [5.30] | 19.95 [4.60] | 21.15 [6.20] | 19.20 [4.85] | 0.180 | 0.198 |
| Venous PO₂, mmHg | 51.90 [18.60] | 56.60 [21.98] | 56.60 [12.05] | 63.94 [25.60] | 0.068 | 0.089 |
| Maternal age, years | 32.99 ± 6.78 | 30.52 ± 7.45 | 37.25 ± 3.11 | 33.39 ± 5.84 | 0.020 | 0.031 |

Values are mean ± SD for approximately symmetric variables and median [IQR] for skewed variables.

Table 3. Categorical variables by four-category discharge disposition

| **Variable coded as yes** | **Healthy discharge (n=177)** | **DAMA (n=62)** | **Transfer (n=8)** | **Death (n=23)** | **P value** | **FDR q** |
| --- | --- | --- | --- | --- | --- | --- |
| SGA status | 51 (28.8) | 16 (25.8) | 2 (25.0) | 9 (39.1) | 0.686 | 0.914 |
| Male sex | 103 (58.2) | 35 (56.5) | 4 (50.0) | 14 (60.9) | 0.950 | 0.990 |
| Cesarean delivery | 143 (80.8) | 49 (79.0) | 7 (87.5) | 19 (82.6) | 0.990 | 0.990 |
| Maternal hypertension | 18 (10.2) | 4 (6.5) | 1 (12.5) | 6 (26.1) | 0.071 | 0.383 |
| Gestational diabetes | 29 (16.4) | 13 (21.0) | 0 (0.0) | 4 (17.4) | 0.565 | 0.904 |
| Premature rupture of membranes | 16 (9.0) | 3 (4.8) | 2 (25.0) | 0 (0.0) | 0.096 | 0.383 |
| Chorioamnionitis | 3 (1.7) | 0 (0.0) | 0 (0.0) | 1 (4.3) | 0.314 | 0.815 |
| Urinary tract infection | 4 (2.3) | 0 (0.0) | 0 (0.0) | 1 (4.3) | 0.407 | 0.815 |

Percentages are within outcome category

Table 4. Secondary logistic model for SGA classification

| **Predictor** | **OR** | **95% CI** | **P value** |
| --- | --- | --- | --- |
| Gestational age, per week | 1.11 | 0.97 to 1.26 | 0.138 |
| Platelets, per 50×10⁹/L | 0.71 | 0.59 to 0.86 | <0.001 |
| Red blood cells, per 1×10⁶/μL | 0.57 | 0.38 to 0.86 | 0.007 |
| Venous PO₂, per 10 mmHg | 1.13 | 0.98 to 1.30 | 0.091 |
| Maternal hypertension | 2.21 | 0.96 to 5.13 | 0.064 |
| Gestational diabetes | 0.64 | 0.28 to 1.46 | 0.286 |

Complete-case N was 270. Model AUC was 0.704 and Brier score was 0.180.

Table 5. Exploratory RBC and PO₂ interaction model and Firth robustness analysis

| **Analysis** | **Predictor** | **OR** | **95% CI** | **P value** |
| --- | --- | --- | --- | --- |
| Interaction model | Gestational age, per week | 1.08 | 0.94 to 1.24 | 0.262 |
| Interaction model | Platelets, per 50×10⁹/L | 0.72 | 0.59 to 0.87 | 0.001 |
| Interaction model | Red blood cells, per 1×10⁶/μL | 0.19 | 0.05 to 0.71 | 0.014 |
| Interaction model | Venous PO₂, per 10 mmHg | 0.45 | 0.16 to 1.28 | 0.134 |
| Interaction model | Maternal hypertension | 2.17 | 0.93 to 5.08 | 0.075 |
| Interaction model | Gestational diabetes | 0.62 | 0.27 to 1.43 | 0.262 |
| Interaction model | RBC and PO₂ interaction term | 1.23 | 0.97 to 1.55 | 0.082 |
| Firth sensitivity | Gestational age, per week | 1.10 | 0.97 to 1.26 | 0.141 |
| Firth sensitivity | Platelets, per 50×10⁹/L | 0.72 | 0.59 to 0.87 | <0.001 |
| Firth sensitivity | Red blood cells, per 1×10⁶/μL | 0.58 | 0.39 to 0.86 | 0.006 |
| Firth sensitivity | Venous PO₂, per 10 mmHg | 1.12 | 0.98 to 1.30 | 0.089 |
| Firth sensitivity | Maternal hypertension | 2.19 | 0.95 to 4.98 | 0.064 |
| Firth sensitivity | Gestational diabetes | 0.66 | 0.28 to 1.44 | 0.305 |

The likelihood-ratio test for adding the interaction term to the noninteraction model yielded p=0.073..

Table 6. Conditional RBC association across venous PO₂ values from the exploratory interaction model

| **Venous PO₂, mmHg** | **RBC OR** | **95% CI** |
| --- | --- | --- |
| 38.3 | 0.42 | 0.24 to 0.73 |
| 44.7 | 0.48 | 0.30 to 0.77 |
| 53.8 | 0.58 | 0.38 to 0.88 |
| 63.9 | 0.71 | 0.44 to 1.15 |
| 74.3 | 0.88 | 0.47 to 1.65 |

These conditional estimates are exploratory because the interaction term itself was not statistically significant.

Table 7. Candidate primary models and internal validation

| **Model** | **Complete-case N** | **Events** | **AIC** | **Corrected AUC** | **Corrected Brier** | **Corrected calibration slope** | **Selection frequency** |
| --- | --- | --- | --- | --- | --- | --- | --- |
| Gestational age plus SGA | 208 | 31 | 157.06 | 0.71 | 0.11 | 1.00 | 0.016 |
| Gestational age plus logCRP plus pH | 208 | 31 | 147.41 | 0.78 | 0.11 | 0.92 | 0.546 |
| Gestational age plus SGA plus logCRP plus pH | 208 | 31 | 149.36 | 0.77 | 0.11 | 0.88 | 0.072 |
| Gestational age plus SGA plus logCRP plus pH plus RBC | 208 | 31 | 149.34 | 0.79 | 0.11 | 0.86 | 0.366 |
| Selection-aware model-selection process | 208 | 31 |  | 0.77 | 0.11 | 0.85 |  |

Likelihood-ratio p values were 0.0029 for adding logCRP and pH to the gestational-age plus SGA model, 0.841 for adding SGA to the reduced physiology model, and 0.155 for adding RBC to the combined model. Bootstrap resampling used 1000 iterations.

Table 8. Sensitivity analyses for alternative birth-size coding and DAMA handling

| **Analysis** | **Predictor** | **OR** | **95% CI** | **P value** | **Corrected AUC** | **Corrected Brier** | **Corrected calibration slope** |
| --- | --- | --- | --- | --- | --- | --- | --- |
| Primary endpoint with z score | Gestational age, per week | 0.74 | 0.61 to 0.89 | 0.002 | 0.78 | 0.11 | 0.89 |
|  | Birth-weight z score | 0.86 | 0.62 to 1.20 | 0.373 |  |  |  |
|  | logCRP | 1.55 | 1.04 to 2.32 | 0.031 |  |  |  |
|  | Venous pH, per 0.1 increase | 0.48 | 0.27 to 0.87 | 0.016 |  |  |  |
| Nonhealthy composite | Gestational age, per week | 0.91 | 0.81 to 1.02 | 0.115 | 0.57 | 0.22 | 0.78 |
|  | SGA status | 0.89 | 0.50 to 1.58 | 0.688 |  |  |  |
|  | logCRP | 1.41 | 1.08 to 1.85 | 0.012 |  |  |  |
|  | Venous pH, per 0.1 increase | 1.01 | 0.70 to 1.45 | 0.956 |  |  |  |
| Best-case DAMA healthy | Gestational age, per week | 0.73 | 0.61 to 0.88 | <0.001 | 0.79 | 0.09 | 0.91 |
|  | SGA status | 1.15 | 0.46 to 2.83 | 0.767 |  |  |  |
|  | logCRP | 1.65 | 1.14 to 2.40 | 0.008 |  |  |  |
|  | Venous pH, per 0.1 increase | 0.44 | 0.25 to 0.79 | 0.006 |  |  |  |

The likelihood-ratio p value for adding birth-weight z score to the primary reduced physiology model was 0.377. The likelihood-ratio p value for adding SGA to the best-case DAMA model was 0.768.

Table 9. Exploratory reduced multinomial model

| **Outcome comparison against healthy discharge** | **Predictor** | **Relative risk ratio** |
| --- | --- | --- |
| DAMA | Gestational age, per week | 1.03 |
| DAMA | SGA status | 0.78 |
| DAMA | Red blood cells | 0.95 |
| DAMA | Venous pH, per 0.1 increase | 1.16 |
| DAMA | logCRP | 1.19 |
| DAMA | Venous PCO₂, per 10 mmHg | 0.77 |
| Transfer | Gestational age, per week | 0.79 |
| Transfer | SGA status | 0.47 |
| Transfer | Red blood cells | 0.61 |
| Transfer | Venous pH, per 0.1 increase | 1.02 |
| Transfer | logCRP | 1.98 |
| Transfer | Venous PCO₂, per 10 mmHg | 1.93 |
| Death | Gestational age, per week | 0.76 |
| Death | SGA status | 1.23 |
| Death | Red blood cells | 0.61 |
| Death | Venous pH, per 0.1 increase | 0.44 |
| Death | logCRP | 1.55 |
| Death | Venous PCO₂, per 10 mmHg | 0.77 |

Global drop-one likelihood-ratio tests for this exploratory model were p=0.040 for gestational age, p=0.690 for SGA status, p=0.602 for red blood cells, p=0.161 for venous pH, p=0.062 for logCRP, and p=0.052 for venous PCO₂. Multiclass Brier score was 0.474. One-vs-rest AUC values were 0.631 for healthy discharge, 0.648 for DAMA, 0.824 for transfer, and 0.818 for death, with macro-average AUC 0.730. Category-specific confidence intervals are intentionally not presented because of sparse class sizes.
